# Embodiment, Relationships, and Sexuality: An Ethical Analysis of Extended Reality Technologies

**DOI:** 10.1007/s11948-025-00563-y

**Published:** 2025-11-14

**Authors:** Erick José Ramirez, Laura Clark, Sydney Campbell, Julian Dreiman, Dorian Clay, Raghav Gupta, Shelby Jennett

**Affiliations:** 1 Philosophy Department, Santa Clara University, Santa Clara, USA; 2 Santa Clara University, Santa Clara, USA; 3 Brain and Computer Sciences, MIT, Boston, USA; 4 Accenture, Chicago, USA; 5 Carnegie Mellon University, Pittsburgh, USA; 6 Harvard University, Cambridge, USA

**Keywords:** Applied ethics, Augmented reality, Embodiment, Extended reality, Relationships, Virtual reality

## Abstract

Communication technologies change the way we relate to each other and ourselves. In this essay we analyze the effects that extended reality (XR) technologies are likely to have on conceptions of the self, romantic relationships, and other associated concepts like sexual orientation. While these technologies are in their infancy, key psychological and philosophical concepts are already being explored. We begin by defining extended reality and the family of technologies that make it possible. We pay special attention to the way these immersive technologies ground the experiences of presence which can become virtually real. These experiences provide a useful framework for understanding the phenomena of XR embodiment. XR embodiment, the experience of one’s self as embodied in XR, opens up the possibility of blended physical and digital narrative selves which form the basis of new forms of relationships. In a future where XR is incorporated into the basic social and political structures of society, XR embodiment and virtually real experiences challenge normative concepts like sex and sexual orientation. Contemporary conceptions of the self, sex, consent, and love emerged in purely physical contexts to help us navigate the limitations of physical embodiment. XR embodiment requires a new ethical framework to make room for these possibilities. We end the paper by assessing ethical risks XR embodiment can introduce for XR developers, and researchers.

## Introduction

Technological developments, especially in the communication sector, have a way of changing how people relate to one another. People integrate new technologies into their interpersonal, and even romantic, relationships almost as soon as the technologies become available. In 1965, a time when computational technologies were almost exclusively used by governments and corporations, students at Harvard launched “Operation Match.” They intended to bring together romantic partners using the power of the Avco 1790 computer which processed data from a questionnaire (Matthews, 1965).

Several years later another group of Harvard students, including Mark Zuckerberg, would launch “TheFacebook,” with the intention of helping students at Harvard more easily connect with one another. TheFacebook would eventually morph into “Facebook” and then quickly become the world’s largest social media platform. Social media platforms like Facebook, Instagram, WeChat, and Tiktok have profoundly changed how people interact with one another, how they develop and conceive of relationships, and even altered how politicians and corporations communicate (Ledbetter, 2016; Vallor, 2012).

By the 21st century romantic relationships have changed in light of social media and other “cyber” spaces. Developers of online data websites have found new avenues for connection in apps like Bumble, Hinge, and Tinder. Dating websites even bring together people of specific religious, ethnic, or racial identities and exist for those looking to have discreet affairs. Philosopher Aaron Ben-Ze’ev, speaking on the rising popularity of online dating sites, argued that such platforms have become so popular because:

It takes less effort to find romantic partners in cyberspace than at bars, shopping malls, or supermarkets…meeting online has become the most popular way couples meet, eclipsing meeting through friends for the first time around 2013. (Ben-Ze’ev, 2021)

As Ben-Ze’ev notes, web-based technologies have led to large-scale cultural rethinking of romance (McArthur, 2022; McArthur & Twist, 2017; Nyholm et al., 2022). They have also forced revision of the moral and legal environments surrounding interpersonal relationships, including the laws and norms relating to sexual harassment (Barak, 2005; Kennedy, 2021). In addition to the proliferation of these social media platforms and dating sites, virtual and augmented reality technologies have recently entered the commercial marketplace. These technologies extend the realm of possibility for virtual connection. Because XR devices are now commercially available, it’s possible that we’ll spend more of our personal, economic, and political lives in XR spaces.

In this article, we examine the potential these extended reality (XR) technologies have to change our concepts of the self and its connection to the body. Can contemporary theories of identity make room for identities that blend physical and XR elements? We argue that current theories of identity can make room for forms of identity that blend physical and XR embodiment. If we’re right, then these blended identities create strain on pre-existing concepts because such norms will no longer easily apply to these new contexts. This is especially true for conceptions of sexual orientation, romantic relationships, and consent because they were originally constructed to function in purely physical spaces and contexts.

We begin (Sect. “Extended Reality”) by explaining the nature of XR technologies to argue that they can enable virtually real experiences in users (i.e., experiences which are felt and treated as if they were happening in physical space). We then (Sect. “Embodiment”) contrast XR embodiment against physical embodiment to suggest that one’s sense of self in technologically mediated spaces can incorporate both physical and XR bodies to produce hybrid conceptions of the self (e.g., a sense of who we are that includes aspects of physical and XR bodies).

Having provided evidence to show that XR embodiment can produce blended forms of identity, we then, in the proceeding sections, argue that blended embodiment— the experience of identifying with aspects of one’s physical and XR body simultaneously—will strain current understandings of identity-related concepts. These include concepts of the self (Sect. “Identity”), sexual orientation (Sect. “Sex & Orientation”), and romantic relationships (Sect. “Relationships in XR”)—concepts originally developed to function solely in the context of physical embodiment. This strain will also require us to reevaluate the moral concepts associated with identity and relationships, especially the moral frameworks developed around consent (Sect. “Consent”). In the final section of the paper (Sect. “Evaluating XR Applications”) we argue that anyone interested in developing or evaluating simulations in extended reality (e.g. institutional review boards, developers, etc.) must take into account not only the possibility that XR research may harm subjects by exposing them to virtually real experiences, but also introduce risks associated with morally problematic forms of embodiment.

## Extended Reality

Commercially available XR technologies have a relatively short history. In 2016, Oculus (owned by Facebook’s parent company Meta) and HTC both launched the first widely available commercial virtual reality devices. Microsoft began selling its augmented reality (AR) device, the HoloLens, in the same year. More recently, Apple introduced its own AR headset in 2023, the Vision Pro. These hardware devices share important similarities and differences and represent distinct points along the XR spectrum.

When we discuss XR technologies, we aim to capture a wide range of technologies. The core feature XR technologies share is that they introduce simulated content as part of a user’s experience of the world.^1^ The XR spectrum includes technologies already in widespread use, including digital filters used on social media platforms like Snapchat and TikTok. These filters allow users to apply real-time alterations to their faces and bodies while recording a video. Filters overlay new digital content in such a way that they create a composite image consisting of physical features of the original environment along with digital elements that modify those features. AR technologies operate very much like filters in that they alter a user’s experience of the world by either overlaying digital content onto it or by removing content. As examples of both uses, consider an AR mapping tool that gives users turn by turn directions (overlaying information directly within their field of vision) or AR ad blockers that identify billboards and other advertisements in physical spaces and replace them with non-commercial images or remove them from a user’s experience altogether. While digital filters have been commonplace for some time, AR content is still working its way into everyday life, with certain games and platforms like Pokemon Go and Snapchat being popular use cases.

Filters and AR technologies occupy one side of the XR spectrum. Virtual reality (VR) technologies occupy the other. Unlike AR technologies, which add or subtract digital content to a user’s experience of the physical world, VR technologies completely replace a user’s experience of the world with a new digital environment that bears little to no relation to the physical environment the user is in. Importantly, XR technologies across the spectrum are known for being immersive, which allows them to engage users in ways that other media technologies do not and which are responsible for XR’s unique ability to complicate experience. In what follows, we focus our analysis on how XR hardware generates virtually real experiences and why virtually real experiences of embodiment are important.

XR technologies have generated a lot of interest from researchers. They are known for having unusually powerful impacts on users through the creation of unique forms of experience. While it is possible to become engaged while reading a book or get wrapped up in a film to the point of yelling at the characters on screen, VR and AR are unique in their ability to take immersion to a new level. XR technologies are thought to do this by generating an experience of “presence” (Cummings & Bailenson 2016; Sanchez-Vives & Slater, 2005). Presence, in this context, refers to at least one of two possible experiences: one is the experience of *being located in* (or being “present” in) a simulated environment instead of the physical environment a user is in, and the other is the experience of being able to *actually do things* in a simulated environment. Maria Sanchez-Vives has referred to these as “being there” and “doing there” forms of presence (Sanchez-Vives & Slater, 2005).

Some have argued that XR technologies, given the right circumstances, are also able to generate virtually real experiences (Montefiore & Formosa, 2022; Ramirez, 2022). These experiences extend beyond presence as being-there or doing-there and, as the name suggests, include the idea that users undergoing a virtually real experience are treating that experience as more than a fictional or simulated one. According to Ramirez (2022),

[a] virtually real experience, like a highly present experience, involves a kind of successful illusion. Where a present experience requires successfully having a user believe they’re located inside a virtual space (even if only temporarily), virtually real experiences require successfully having users believe that virtual events and virtual objects are real events or real objects and for their bodies to respond appropriately (even if only temporarily). Virtually real experiences, we can say, are those experiences which, while in the grip of the simulation, are responded to (physiologically, psychologically, behaviorally) *as if* they were real experiences. (Ramirez, 2022)

To see the differences between presence and virtually real experience, imagine a user playing a combat-oriented game in XR. Such a user may have the experience of being located in the game world, face to face with whatever monsters they are battling, and they may also feel like they’re able to interact realistically with that world (e.g., physically reaching over to grab a weapon). They would be fully present in the being-there and doing-there senses. So long as the user is actively enjoying this experience, it’s unlikely that they’re also having a virtually real experience of these events. A person actually dropped into the fight-or-flight situations typical of games like these is unlikely to enjoy the experience in the way gamers do (Zendle, Kudenko, & Cairns 2018).

Contrast this user with a user in the middle of a session of virtual reality exposure therapy (VRET) set in a virtual therapist’s office (Parsons & Trost, 2014). Attempting to learn therapeutic strategies to combat a phobia of spiders, the user is gradually exposed to ever more realistic depictions of spiders while practicing cognitive and behavioral techniques. Such a user may also feel present in the being and doing-there senses, however, they may also evince behaviors consistent with having virtually real experiences in that setting. If, when exposed to virtual spiders, they respond (cognitively, behaviorally, physiologically, etc.) similarly to how they would to a physical spider, then it becomes clearer that the user not only feels present but also as if they were really being exposed to spiders. Virtually real experiences have been reported by users across varied XR simulations, including social ones, where users sometimes report feeling a “phantom touch” when others interact with their virtual bodies (Zhang et al., 2022). Such experiences play an important role in the experience of embodiment in XR and we turn our attention to such experiences next. In section three we provide evidence that virtually real experiences are also possible when individuals are embodied in XR simulations.

## Embodiment

We have argued that XR technologies, under the right conditions, can produce virtually real experiences in people. These create an array of ethical and metaphysical questions and have begun to receive serious study (Chalmers, 2022, Jacobs & Anderson 2019; Montefiore & Formosa, 2022; Ramirez, 2022). In some cases, users can experience the events occurring to them as if they were occurring in the physical world. Early evidence suggests that this phenomenon extends to a person’s experience of their body. Here, we turn our attention to these experiences.

We begin by distinguishing between two forms of embodied experiences: experiences of physical embodiment and experiences of XR embodiment. We do this in order to show not only that people can feel a sense of ownership over (and identity with) their XR bodies, but also that psychological conflicts between physical and XR embodiment can cause real dysphoria.^2^ In this section we’ll suggest that the best evidence we have about XR embodiment suggests that the concept of the self, of what constitutes who someone is, is likely to include increasingly blended forms of embodiment in these environments. If we’re right, these new blended identities require a reorientation of ideas of the self and of relationships because the concepts of self and relationships developed to make sense of purely physical embodiment do not extend well to these new environments.

Let’s begin with the experience of physical embodiment. The self and the physical body are, for most of us, intimately connected, and physical embodiment is one way of understanding that relationship. When we use the phrase “physical embodiment,” we mean to specifically refer to the experience of the self (a person’s sense of having and being a self) as that self is connected to a person’s physical body. The idea that the way that a being understands itself or the world is determined, or even constituted, by how that being is embodied is not new. Theories of embodied cognition have been articulated and defended for some time (Barsalou, 1999; Clark, 1997; Glenberg, 1999).^3^ In relation to XR, this idea has surprising implications. If we understand the self through embodied experience, and if XR can affect our experience of embodiment, then we suggest XR can affect how we understand the self. Some theorists have argued that the sense of a self might even originate from the activity of embodied proprioceptive (Parsons & Neisser 2024) or interoceptive mechanisms (Seth, 2019).^4^

Equally importantly, humans have a near-universal tendency to use the technologies available to them to express the self through the body (Blanco-Dávila, 2000).^5^ Choices of clothing, hairstyle, makeup, colored contact lenses, the use of exercise or diet routines, and cosmetic surgeries are all ways that people modify physical bodies to express the self through it. Body modifications, especially surgical alterations, for self-expression have received significant attention from ethicists, though this attention is largely focused on setting ethical limits on who can modify a body or for what purpose (McCabe et al., 2017; Sarajlic, 2020). Although physical bodies are often how the self is expressed, the body itself also places limits on how far it may be altered. In other words, there are biological limitations on how far the body may be stretched, augmented, or nullified before vital internal functions become impossible to sustain. XR technologies promise to radically expand the possibilities for embodiment and expression.

If physical embodiment is a way of understanding how individuals experience the self through their physical bodies, XR embodiment is a way of referring to a similar, albeit technologically mediated, experience. The XR spectrum encompasses a wide range of hardware, and so the opportunities for embodiment vary. XR embodiment includes the use of filters to change someone’s appearance and thus how they experience being embodied. Filters can overlay a varied set of bodily alterations ranging from the cosmetic (e.g., blemish removal) to complete overhauls of one’s appearance (e.g., changing facial structure or overlaying a digital head or body to better reflect a user’s self presentation). XR embodiment extends beyond filters to include other ways that users can be embodied.

Physical and XR embodiment are both ways of experiencing the self through a body. However, these forms of embodiment differ. We can only physically modify bodies so far before they cease to function and a person dies. Certain technologies can extend these limits but biological limitations will always be a part of the experience of physical embodiment.^6^ Comparatively, XR embodiment imposes fewer restrictions on embodiment.^7^

Studies of avatar customization and XR show that most people use XR to embody themselves using idealized versions of their physical bodies, much as people use beauty filters to create idealized images and videos of themselves (Freeman et al., 2020). XR embodiment, however, also allows users to express themselves in more radical ways. XR embodiment allows someone to embody themselves as an animal, a cloud of color-changing mist, imaginary alien forms, or even as a favorite celebrity.^8^ Some restrictions on embodiment remain, however, as the owners of virtual spaces have control over the options their users can select. For example, Meta, the company behind the creation of the social XR platform known as *Horizon Worlds*, has worked to reverse its decision to forgo legs for user avatars (Welch, 2023). Their previous decision barring users to embody themselves with legs placed an obvious limitation on XR embodiment and one that restricted self-expression, especially for those with mobility-related disabilities (Zhang et al., 2022).

Because XR technologies are so immersive and because they enable users to have virtually real experiences, users have reported that XR embodiment is sometimes transformative. Such experiences can result in a unique conflict between physical and XR embodiments. Guo Freeman and colleagues investigated how users experienced embodiment in VR avatars. Many of Freeman’s subjects expressed a strong connection with their XR bodies:

As P22 (Male, 32, white) explained, he felt that his avatar in social VR was “truly an extension” of himself: “With rec room, when you’re creating the avatar, you’re actually looking at it and you can move around and turn around. It’s truly an extension of you. If it’s in a normal game, it’s not as engaged. I’ll just find the first thing that’s like kind of okay and go with it.” (Freeman et al., 2020).

One interesting result was that “participants mentioned that their avatars in social VR helped them explore other undiscovered potentials of themselves” (Freeman et al., 2020). In some cases, the exploration of XR embodiment produced radical confrontations between XR and physical bodies. Two of Freeman’s subjects discuss their experiences:

Using a feminine avatar makes me confident not only in VR but also in real life. I feel like that would be actually more real than the real you in real life. Because in real life, you’re stuck with what you were born with. But in VR, you can be what you truly feel like you are inside. This experience actually gave me confidence to start my [transgender] procedure in the real life.” (Freeman et al., 2020).

[B]y using a feminine female avatar, I found that I was just more comfortable with that body, and it’s kind of what I learned about my identity. That was the evidence to myself to consider which direction I wanted to take my actual body outside of the VR. If I found I was happy in VR about my body and I was not happy with my body outside of the VR, why not change it?” (Freeman et al., 2020).

In these cases, until their experiences of XR embodiment, it appears Freeman’s subjects had felt their physical embodiment as connecting to an undesired male identity. As a result of their new ability to become embodied in XR, an aspect of their understanding of their identity changed. XR embodiment, for these subjects, was experienced as female. In this case we have a seeming conflict between physical and XR embodiment. The possibility for conflict between XR and physical embodiment can, as it does in these two cases, have important implications for people.^9^ In the end these specific subjects chose to respond to the conflict between physical and XR embodiment by modifying their physical bodies to look more like their XR bodies.

XR and physical embodiment can come into conflict in other ways. The COVID-19 pandemic of the 2020s caused formerly physical activities (e.g., work, education, entertainment) to happen in virtual environments. Platforms like Zoom became dominant for work- and school-related tasks in many places. Embodiment in these environments, as we’ve argued, is more flexible than in purely physical spaces, and gave many people their first long-term experiences of XR embodiment.^10^ Conflicts between physical and XR embodiment have led some mental health practitioners to propose the adoption of a new variant of body dysmorphic disorder, “Zoom dysmorphia” (Ramphul, 2021).^11^ While conflicts between XR and physical embodiment are beginning to receive attention, pathologization of this conflict is only one way to respond to it. In section four, we investigate how well current theories of identity can make room for identities that include blended embodiment. Blended identities are likely to become increasingly common and it is *blended* identities that are poised to cause the most strain to conceptions of sexual orientation, consent, and intimate relationships.

## Identity

Physical and XR embodiments presuppose a conception of “the self” that is strongly tied to the body. In this section we survey theories of the self to argue that blended bodies can be understood as a genuine part of one’s self. In doing this we sidestep historical debates philosophers have had about the metaphysics of selfhood. Traditionally, philosophers focus on what they call “the reidentification question” (e.g., how can we know that the person in front of us now is the same person we met a year ago?). The focus of these theories has thus been to answer questions about the conditions under which identity holds steady *over time* (Parfit, 1971; Perry, 1972; Lewis, 1976).^12^

Philosophers have tended to focus on *this* question about identity because of its connections with moral and legal responsibility. For example, to hold someone responsible for a crime, they must be *the same person* who committed the crime. Philosophers then ask: what must be true about the person being accused and the person who committed the crime in order for us to say that they are the same person?

While the reidentification question has a rich and interesting history, we focus on a different question relating to identity philosophers refer to as “the characterization question”: under what circumstances is something (a body part, an event, a memory) a legitimate part of a person’s self? This question centers on who or what we take ourselves to be and under what conditions we can be wrong about who we think we are (Schechtman, 1996). We focus on characterization, not reidentification, because of our interest in determining whether and to what extent XR bodies can be accounted for as a part of a person’s genuine or authentic self.^13^

We have argued that XR embodiment affects a person’s conception of who they are in terms of both body ownership (physical and XR) and characterization (whether the real self most closely aligns with one’s physical body, one’s XR body, or a blend of the two). In this section, we argue that some contemporary theories of identity lend support to the claim that XR bodies are a part of people’s genuine selves. In sections five through seven we explore the implications of these conclusions. XR embodiment, if taken seriously, raises important questions about the nature of sex, sexual orientation, and relationships.

Technology has already complicated traditional conceptions of whether the self includes only physical or biological components. Biotechnologies create questions about the moral and legal status of prosthetics. The law recognizes a difference between damage done to one’s property and damage done to one’s body. However, are prosthetics a part of a person’s body such that damaging a prosthetic amounts to physical assault, or are prosthetics more like property? Writing about the moral implications of prosthetic embodiment, Aas (2021) argues that:

the things that social institutions ought to protect, in this bodily way, are those things that are critical to our functioning as equals in our actual social world. And, I will argue, once we clear away naturalistic fallacies regarding what can and cannot be a ‘body part’, we will see that equality among functionally diverse embodied beings like us requires recognition of bodily status for many artificial and inorganic items. (Aas, 2021)

Aas’ argument is complex. Bodies receive special protection in the law, according to Aas, at least partially because they are what allow people to function socially in the world. If prosthetics are important for allowing people to engage in typical social functions, then prosthetics should be treated more like body parts than personal property. For Aas, something can become a part of body if all of the following conditions are met (Aas, 2021):

1. they give our minds a toehold in the world.
2. we feel profoundly attached to them, and violated if they are invaded.
3. we rely on them to do what we want in the world.

A subjective sense of ownership (condition 2) doesn’t by itself suffice to justify something as a part of a person’s body (i.e. someone’s beliefs about body ownership may be sincere but delusional or mistaken). It matters that all three conditions are met. This argument can be extended to XR embodiment. In a future where our personal and professional lives are lived in XR, XR bodies become important to function in social settings. XR bodies would give our minds a toehold into such a world and we have already shown that people can feel profoundly attached to them. Aas’s account of embodiment and the conditions under which something should be properly understood as being a part of a person’s body show that XR bodies should be included in conceptions of embodiment.

Narrative theories of identity offer another way to explain the relationship between physical embodiment, XR embodiment, and the self. Narrative theories of identity were specifically introduced in order to address the characterization question and thus we pay specific attention to them in this section. Philosopher Schechtman (2012) explains the purpose of a narrative theory:

According to the narrative approach the identity of a person is narrative in structure, and the individuation of persons is indexed to the unity of single ongoing narratives…We constitute ourselves as persons, I argue, by coming to understand our lives as narratives with the form of the story of a person’s life. Having a narrative of this sort is instead a way in which persons implicitly organize their experiences and undertake their deliberations. A person experiences what happens in the present in light of what has come before and what is expected or planned for the future, and in this way temporally remote times are brought into present experience. (Schechtman, 2012)

Narrative theories explain the characteristics of living a recognizably human life in terms of life stories. These are relational and interdependent (Lindemann, 2001), i.e., a person’s narrative is authored by the person but also informed by the narratives others may have of them. Narrative theories of identity address the characterization question by highlighting the fact that to make sense of who a person is we need to tell or be told a story about who they are. That story will include historical facts about the person (e.g., where they were born, grew up, etc.). It should also include first-personal information about how they understood their life and their relationship with their bodies and other people (e.g., the narrative of a person suffering from alien hand syndrome will include narrative elements describing a very different relationship between the self and physical embodiment from the narrative of someone without this condition).

However, just as Aas was concerned that a person may have a delusional or mistaken sense of body ownership over something, narratives may be similarly problematic. Schechtman places two constraints on narratives that help us understand when a narrative accurately tells the story of who someone is:

The two main constraints are the “reality constraint”, which says that someone’s narrative must conform to fundamental and largely uncontroversial everyday facts about the nature of the world we live in (e.g., humans do not usually live more than 300 years; they cannot get from Chicago to Paris in less than 4 s; they cannot be in two places at one time), and the “articulation constraint” which says that someone must be able to articulate parts of her narrative locally when appropriate. (2012)

Writing about internet simulations like *Second Life*, Schechtman claimed that the narrative self- constitution theory (NSCV) can explain how the avatars people engage with others through while in those environments can be proper parts of a life narrative —a narrative that includes both physical and virtual components:

NSCV does, however, give us the basic contours of a view of identity that takes seriously the claims that virtual worlds can be a genuine part of a person’s life, and her avatar a genuine part of herself, while still appreciating the very obvious and important differences between [virtual bodies] and [physical bodies]. At the same time, application of this view to the context of virtual worlds like [Second Life] provides a great many insights into what a satisfying narrative view of identity must look like. (2012)

Given the way that XR allows for seamless and immersive interaction between physical and XR bodies, the case for XR bodies to be properly included in the context of a person’s life narrative is even stronger than it is for avatars in non-immersive simulations like *Second Life*. Users, like those in Freeman’s study, can feel a strong sense of ownership over their XR bodies and have virtually real experiences of interacting with those bodies and others.

There are multiple ways to arrive at the conclusion that XR bodies should be taken seriously as a part of the self. Aas’ (2021) view takes seriously the fact that prosthetics deserve protection not only because people feel ownership over them but because they’re important to functioning as equals in society. XR bodies may soon fill that role in XR environments. Similarly, narrative conceptions of identity support the view that a person’s construction of who and what they are, what they do, and where they’ve been can justifiably incorporate XR bodies and environments as part of a whole-life narrative. While this view is open to critique, we believe the evidence we’ve marshaled supports the claim that XR bodies can be viewed as proper parts of a person.

While a potentially interesting conclusion on its own, in sections five through seven we argue that if XR embodiment should be taken seriously as a proper part of the self then it strains contemporary concepts associated with sex, orientation, consent, and relationships.

## Sex & Orientation

When we discuss sexual orientation we reference both sex and gender. Both are incorporated into contemporary theories and these concepts have changed in the 20th and 21st centuries, especially in Western contexts (Schudson, 2021). In this section we argue that while the concepts of sex and orientation have changed, these changes focus largely on interpretations of the physical body. Whether sex is understood as a biological or natural kind (Hammer et al. 1993; Stock, 2022), a social construct (Butler, 1990), or a hybrid of these positions (Fausto-Sterling, 2019), the concept of sex is discussed in physical terms (or in thoughts about physical bodies).^14^ Similarly, although gender is widely understood as picking out a constructed set of norms that tie sex to identity, concepts of gender identity and expression are often tied to relationships between individual identity and physical embodiment. Concepts tied to sex and gender, like sexual orientation, follow a similar logic in terms of physical embodiment.^15^ XR embodiment strains traditional concepts of sex and orientation because it creates new possibilities for blended identities not constrained by the biological limitations of physical embodiment.

The concept of sexual orientation has historically been theorized in terms of physical markers. One such marker takes sex as a biological category and gender as a socially constructed one (Muehlenhard & Peterson, 2011). On such views, sexual orientation is biologically tied to sex. Philosopher Stock (2022) calls this approach the “Orthodox account:” where sexual orientation “causes a person to be sexually attracted to…those people of a particular Sex” and is dictated by “the Sex of the desiring subject” and “the Sex of the type of person typically desired by the subject” (Stock, 2022). Though often used as a foundation for constructing theories of sex and orientation, the Orthodox account has met growing criticism throughout the 21st century. This is in large part due to its reductive explanation of sexual orientation (González Vázquez, 2022).

Sexual orientation has also been described variously as based on individual choice (Wilkerson, 2009; Díaz-León, 2017), as not a choice at all but instead as a conceptual act (Ayala, 2017), or as a judgment that an individual makes based on their moral framework or pragmatic goals relating to sex (Christina, 2022).^16^ It is beyond the scope of our argument to take a position on the nature of sexual orientation, especially as the concept is likely to continue evolving and we do not wish to be understood as supporting a specific position on orientation. Our goal in surveying this literature is to convey the variety of positions on sexual orientation. Queer theorists (Rudy, 2022; Pismenny, 2023; Ayala & Vasilyeva, 2015) have suggested that the concept of sexual orientation may require radical restructuring, of amelioration, in light of structural injustices built into it. In that spirit, we argue that XR embodiment is a vehicle (and opportunity) for such restructuring.

XR embodiment challenges current ways of thinking about sexual orientation by fracturing the relationship between the individual and physical embodiment. We have argued that XR makes possible conditions where physical and XR embodiment conflict and where individuals may identify more strongly with XR bodies in response to that conflict. In the documentary *We Met in Virtual Reality* (Hunting, 2022), individuals embody themselves in ways that represent conventional (and unconventional) races, sexes, genders, and species. In the documentary two users, Dustbunny and Toaster, discuss their romantic and sexual relationship and treat these as real experiences even though they occurred in a VR world.^17^

If sexual orientations are understood as strongly tied to physical bodies, then XR embodiment problematizes those conceptions by showing that sexual orientations are less stable than they may appear. XR embodiment also allows for *blended* forms of identity that can include bodies to which traditional sexual orientations may not (and perhaps should not) apply.^18^ Blended identities create conceptual strain for theories of sexual orientation tied strongly to physical markers. Section six explores the possibility, introduced by McArthur (2022) of new forms of sexual orientation like “digisexuality.”

## Relationships in XR

Relationships, especially intimate ones, are inherently complex. XR embodiment and blended identity further complicate these relationships. In this section, we canvas unanswered questions about the intersection of identity, XR embodiment, and romantic relationships. We begin by assessing whether contemporary frameworks of love can make room for XR embodiment and blended identities. We pay special attention to the psychological and physical constraints such theories place on romantic relationships. In connection with this discussion we explore how XR embodiment may require a reanalysis of standard notions of sexual ethics with respect to consent and disclosure.

Theories of romantic relationships traditionally describe love itself as their defining characteristic. Whether love is best conceived as a basic emotion (Shaver et al., 1996), as a set of propositional attitudes directed at a beloved (Solomon, 2010), or as a potentially dangerous “limerent” state (Tennov, 1998) is a matter of dispute. In addition to love, elements like sex, commitment, physical touch, and reciprocity are often tied to romantic relationships. Solomon (2006) even argues that “love” is a social construction dependent upon how societies understand sexual attraction and the emotions that go along with it. What unites most conceptions of love is a link between love and sex. While sex need not actually be realized between lovers (e.g., distance, disability, or social conditions may make consummation impossible or unpleasant), a counterfactual desire for sex is a common criterion:

Romantic love isn’t about sex (a common fallacy) but it depends on sex, thrives upon sex, utilizes sex as its medium, its language and often its primary content. Whatever else it may be, romantic love begins with the inspiration and exhilaration of sexual attraction…sexual attraction is not ‘just physical’ of course…but whatever else it may be, sex is bodily and sexual desire engages us as embodied creatures for whom ‘looks’ and the blessings of nature are at least as important as the egalitarian insistence that we are all, ‘deep down,’ essentially the same. (Solomon, 2006)

Solomon ties romantic love to sex and understands sex as embodied, physical, and focused at least partially on the beloved’s physical appearance. XR embodiment, because it requires us to take seriously the possibility of blended embodiment, is in tension with views like Solomon’s. By treating sex as as an activity that can only happen between physical bodies, these views would not capture virtually real experiences of sex between individuals with XR bodies.^19^

Asexuality and the possibility of asexual romance poses a challenge to this understanding of romantic love. Research on asexuality suggests that individuals who identify as asexual cover a wide-spectrum of beliefs about sex (some asexuals have sex, some do not). Luke Brunning & Natasha McKeever have argued that asexuality presents a powerful challenge to traditional notions of love and embodied sex like Solomon’s:

given sufficient theoretical grounds to describe asexuality as a sexual orientation, then we would have reason to move away from thinking sexual orientations are orientation towards people of a particular biological sex or of erotic experiences as being ‘fundamentally gendered’. Instead, we may end up thinking about sexual orientation in a different way altogether, deprioritising sex and/or gender, and instead focussing on patterns of attraction towards traits, behaviours, situations, or even individuals. (Brunning & McKeever, 2020)

Like asexuality, XR embodiment poses similar challenges to traditionalist accounts of love and sex. Conceptualizations of sex itself may need rethinking in the light of these challenges.

How we think about and count a particular act as a sex-act is predicated on a series of normative questions. For example, we may care about whether someone has had sex with another because it breaks a monogamous commitment they made, because it creates new duties between lovers (like disclosing sexually transmitted infections), or because it violates a social norm about who is permitted to engage in sex at all (e.g., zoophilia) (Christina, 2022; Rudy, 2022). XR embodiment thus strains conceptions of sex that require only physical touch or physical embodiment. Ought XR embodied sex count as sex for these purposes? We argue below for an ameliorative approach to the conceptual strains created by XR embodiment.

The widespread adoption of web-based technologies, especially online dating and social media platforms, has already created confusion about the nature of sex in the context of digitally mediated interactions (McKeever, 2022; Nyholm et al., 2022). McArthur (2022) have argued that digitally mediated romances require us to accept the rise of a new form of sexuality they call digisexuality:

digisexuality can be viewed in terms analogous to how we understand other sexuality-based identities such as kink or non-monogamy. When a sexual practice becomes the basis for an identity, we tend to see people fall along a spectrum, with occasional practice on one end and what we might call identity-integral practice on the other. (McArthur & Twist, 2017)^20^

McArthur and Twist also distinguish between what they call first-wave digisexualities (which rely on web-based interactions between partners) and second-wave digisexualities (which make use of immersive, often haptic, XR technologies). They note the normative concerns that surround sex and digisexuality. For instance, “in first-wave digisexualities there is ambiguity around what constitutes cheating or non-consensual non-monogamy; in a recent study of 810 partnered adults, 50% reported that watching online pornography counted as cheating (Thompson & O’Sullivan, 2016) which means that while half of the participants believed viewing adult film content was cheating the other half did not,” implying a level of ambiguity about how first-wave digisexual activity is understood (McArthur & Twist, 2017). The Internet and social media have already complicated the situation with respect to romantic concepts. If McArthur and Twist’s arguments are right, our ideas about fidelity have already become strained. So-called second wave digisexualities—which include XR—will add additional complications.

## Consent

McArthur and Twist have suggested that concepts of infidelity are strained by first-wave digisexualities. Closely tied to the concept of infidelity is the concept of consent. Consent, like fidelity, cannot easily account for the complexity that XR embodiment adds to relationships. Accounts of consent—the conditions under which consent to something is possible or ethical—vary widely. Much of academic literature on consent focuses on issues in medical ethics regarding the value and nature of informed consent (e.g., what does a patient really need to know and understand in order to consent to treatment?) (Eyal, 2014).

In sexual ethics, views on the nature of consent include seeing it as a form of mutual contractual agreement (Wertheimer, 1996), an embodied “feeling with” another (Anderson, 2022), and also includes various views that argue that consent by itself is insufficient for ethical sex (Alcoff, 2018; Woodward, 2022). In this section we focus our attention on a recent account of consent offered by Lloyd (2021) to help us explain the strain that XR embodiment causes for traditional understandings of consent as a form of mutual agreement. Lloyd’s position represents fairly common assumptions about the nature of consent and obligation. She argues that people have extensive duties of disclosure in order to receive morally permissible consent to sex.

Specifically, Lloyd argues that people have duties to disclose information about themselves relevant to a potential partner’s “dealbreakers.” A dealbreaker is “a feature that is dispositive of someone’s decision to have sex” (2021). For example, if political affiliation is a dealbreaker for someone—i.e. they will only consent to sex with people who share their political values—then you have a duty to disclose your politics to a potential partner if it’s clear that politics is a dealbreaker for them. In Lloyd’s view, dealbreakers are relative to individuals but not limited just to moral concerns or preferences. Consider one of her examples:

Rose meets Jack at the gym in New York City. When they meet, Rose is wearing long blonde hair extensions braided into her natural hair. Upon asking Rose out, Jack mentions that he finds Rose’s hair ‘gorgeous.’ On their date, Rose is wearing a long almost white wig and again, Jack is ‘all over it.’ Rose’s wigs are expensive and she takes pride in being careful about how she applies them, so that when Rose and Jack eventually have sex, her wig stays put. Sometime later, though, Jack sees Rose without her wig and realizes that one of the main things he had liked about her appearance was not real. Things fizzled out soon after. (Lloyd, 2021)

According to Lloyd, Rose has had non-consensual sex with Jack because she ought to have known, given Jack’s enthusiasm about her hair, that not having natural hair was a dealbreaker for him. For Lloyd, Rose’s wig-wearing was a piece of information relevant to Jack’s ability to consent to sex.

This way of framing the norms for valid consent makes it morally objectionable to withhold information about oneself that might be a dealbreaker for one’s partners regardless of the underlying worldview grounding that dealbreaker. More problematically, Lloyd’s view requires people to *undermine* their identities to fulfill their duties of disclosure. Suppose that Sam identifies, and is socially recognized by others, as a woman. Sam’s sex assigned at birth was male but she has spent many years living, and being treated, as a woman. Sam would like to have sex with Kelly but suspects that Kelly’s views on gender are different from her own. Kelly might not view Sam as a woman and this would be a dealbreaker for Kelly. On a view like Lloyd’s, Sam has a duty to not only disclose her own gender identity (as a woman) but also undermine that identity by disclosing to Kelly that she isn’t a woman (in Kelly’s eyes). Additionally, norms of consent like these require Sam to accept that what she is doing, if she doesn’t disclose, is deceptive.^21^,^22^

A similar problem for consent follows as a result of XR embodiment. If José identifies with components of his XR embodiment (e.g., José makes use of AR overlays to alter his appearance as part of an affirmation of his own identity) then José would have to undermine this aspect of himself by disclosing these elements to anyone with a dealbreaker against XR embodiment overlays.^23^ XR embodiment complicates the nature of consent and disclosure just as it complicates our ideas about identity, orientation, and relationships.

Another issue that arises is tied to sexual deception. “Catfishing” is the best known form of technologically mediated sexual deception (Simmons & Lee, 2020). Deception, on most views, makes genuine consent to sex impossible and XR embodiment can enable users to engage in this form of deception (Ramirez et al., 2023).^24^ The wrongness of sexual deception lies in the victimizer’s intent to deceive. Less clear is whether sexual deception occurs in instances where an XR embodied user identifies with their XR body, lacks the intention to deceive, but engages in sexual or intimate relationships with someone for whom XR embodiment is a dealbreaker.

One possibility to respond to the strains XR embodiment creates for these concepts is to *reify* them and rule out XR embodiment as a valid identity. Reification happens when the norms used in physical spaces are imported into XR spaces. A reified concept of sexual orientation rejects the idea that XR embodiment can matter to sexual orientation either because orientation itself is construed as an innate or physical phenomenon (e.g., the Orthodox account) or because the choices or desires important to orientation are construed in physical terms. While reification is possible, it’s not an attractive position.

Conceptions of identity, orientation, and gender have already shifted throughout the 21st century (Pismenny, 2023; Rudy, 2022; Sarajlic, 2020; Schudson, 2021). First-wave digisexuality has caused social and political changes to norms, concepts, and laws about relationships (McArthur & Twist, 2017; Ramirez et al., 2023). Reification of concepts surrounding identity and sexual attraction leaves them unable to encompass these changes and unable to explain the significance of the psychology of XR embodiment and virtually real experiences (Freeman et al., 2020; Ramirez, 2022). Insofar as the concepts in question are intended to help individuals navigate their social, political, and moral lives, reification leaves these concepts unable to serve these important functions (Ayala & Vasilyeva, 2015).

Another response to the strain that XR embodiment creates would be to *eliminate* the strained concepts entirely (Khader, 2019). Call this the eliminativist approach. Eliminativism about the concepts might be justifiable if the concepts themselves were ultimately irredeemably oppressive or unjust. For example, an eliminativist about gender can argue that

gender roles and all their accompanying arbitrary gender norms have provided us with a profoundly pervasive set of (supposedly) ready-made values. When one identifies with the gender binary, one internalizes a set of norms that makes one feel beholden…The enforcement of gender norms, both from within and from without, is limiting our possibilities and keeping us from living authentically. (Eckley, 2022)

The best response in such a case, the eliminativist argues, is to abandon the concept (i.e., to eliminate it from our vocabulary). Given the central place that identity, gender, sexual orientation, consent, and romantic intimacy play in a life narrative, it’s worth exploring whether or not these concepts can be saved before eliminating them. Eliminativism should be a last resort when a concept, initially developed to serve a function in one context, becomes too strained, harmful, or oppressive to salvage.

A third response to conceptual strain is to take an *ameliorative* approach. Amelioration is the idea that the social and political meaning of a concept should change in order to better serve moral goals (Haslanger, 2012, 2020; Jenkins, 2016). Ameliorative theorists understand their project as distinctly normative (i.e., as relative to a set of interests those concepts are meant to advance):

The task before us now is to determine whether it is possible for a concept to shift in its content—for it to have a different partition of logical space as its content—while remaining the same concept…The questions that guide the ameliorator are many. Is this content/partition what we should be thinking and talking about? Is this what we need to think and talk about to carry on meaningfully, rationally, morally in order to answer the pressing questions? (Haslanger, 2020)

Sally Haslanger provides an example of this process in terms of amelioration with the concept of marriage. A once religious-legal-social concept has, in many countries, undergone amelioration so as to include homosexual partnerships (Haslanger, 2020). Homosexual marriages are marriages (and not some other practice) and are recognized as marriages legally and socially despite the fact that historical conceptions of marriage did not make room for them.

Given the important functions that concepts of identity, sexual orientation, and romantic relationships perform, an ameliorative approach makes the most sense in light of the strains that XR embodiment creates. It makes sense that the concepts of identity should take seriously a person’s lived experience and subjective self-determination (subject to the reality and articulation constraints). Ameliorative theories of gender and sexual orientation have already been offered by others to better account for the experiences of trans and nonbinary persons as well as individuals whose orientations don’t neatly map onto Orthodox accounts (Ayala & Vasilyeva, 2015; Rudy 2022). XR embodiment extends the need for amelioration to these and related interpersonal and intimate concepts.

In light of these concerns, in section eight we offer suggestions intended to aid members of research ethics committees (ERCs), clinical practitioners, funding agencies, regulators, and developers of XR applications.

## Evaluating XR Applications

We have argued that XR technologies create opportunities for people to create blended identities. Although research into XR embodiment is in its early stages, it suggests that people can take their experiences of embodiment in XR seriously and that it can impact how a person understands themselves. In the context of assessing research using XR technology, blended identities create new risks that researchers, clinical practitioners, funding agencies, regulators, and members of research ethics committees should become more familiar with in order to better assess the relative risks introduced by XR technologies.

Traditionally, research ethics is guided by three broad principles: respect for persons, beneficence, and justice (National Commission 1978). We find this an amenable starting point for any assessment of the ethics of XR embodiment. To respect persons who are the subject of an experiment is to treat them as autonomous agents capable of understanding and appreciating the risks involved in an experiment and thus capable of consent. Beneficence in research ethics guides researchers to focus on preventing (or minimizing) harm to subjects and, if possible, to maximize benefits. Studies that impose risks to subjects but whose benefits accrue to others require significant ethical scrutiny. Lastly, justice in the context of research is typically understood in the distributive sense: are the risks, harms, and benefits of a study being distributed fairly? XR embodiment raises moral issues relating to autonomy and beneficence (Madary & Metzinger, 2016; Slater 2020; Ramirez 2021).

Virtually real experiences can, on their own, introduce risk in an XR experiment that requires ERC attention. A virtually real experience of falling out of a skyscraper may not result in actual physical injury but could induce stress, anxiety, and trauma on subjects.^25^ Similarly, any XR simulation that replicates an otherwise ethically fraught methodology (e.g., Stanley Milgram’s obedience studies) deserves additional scrutiny if its design is likely to produce virtually real experiences (Slater et al., 2006; Ramirez, 2018).

Blended embodiment creates two additional problems in addition to those created by virtually real experiences. First, because blended embodiment can produce “transformative” experiences — experiences whose effects are impossible to understand, like becoming a parent, prior to direct experience— can complicate the traditional picture of consent in the context of experimental and medical ethics (Paul, 2014; Pettigrew, 2015). Recall that two of Freeman’s subjects had a transformative experience about their gender identity after XR embodiment. It will be difficult, for subjects in an experiment using XR embodiment, to understand and appreciate (and thus to consent) to such experiences in advance. Second, XR experiments that embody subjects as cows in a slaughterhouse (Ahn et al., 2016), as members of different racial or gender identities (Banakou et al., 2016) or in stereotypically overweight or underweight bodies (Piryankova et al., 2014) raise additional ethical questions in connection with the harm such embodiment may cause.^26^ Guo Freeman’s research suggests that the experience of XR embodiment can be transformative —it can alter the relationship a person has with their physical body. Nonconsensually exposing someone to such an experience, especially when it would force someone to become embodied in a body they may object to, requires an especially high level of moral scrutiny.^27^

Anyone interested in safeguarding individuals from exposure to accidental harm or trauma should give additional scrutiny to XR simulations (research, games, social media applications, etc.) that are apt to produce virtually real experiences of embodiment. Recognizing the legitimacy of blended identities, those evaluating XR must acknowledge the technology’s potential to expand or limit a user’s sense of self. In cases where evaluators have reason to believe blended identities exist, they must exercise a similar level of duty and care for the user as if they were operating in an exclusively physical space.

## Conclusion

XR technologies create conditions for experiences that contemporary moral theorizing has left underexplored. Under the right conditions individuals can feel connected to, even sincerely identify with, augmented and virtual reality bodies in ways that strain (or challenge) the relationship between the self and the physical body. We have argued that XR embodiment merits inclusion in any ethical analysis of interpersonal interactions between users in augmented or virtual reality spaces.

Any attempt to carry out such an analysis will have to wrestle with the fact that attempts to integrate both physical and XR forms of embodiment create conceptual strains for existing frameworks. Our concepts of who and what we are, what it means to engage in an intimate relationship, what we are orienting toward in those relationships, and the norms grounding ethical consent in those interactions become strained in these contexts. While we have not argued for a particular positive way of resolving these strains (a project we believe is too complex and interdisciplinary to carry out here), we have argued that amelioration, not elimination or reification, is the best approach to this strain. As more and more of our personal, professional, economic, and intimate experiences are spent in XR environments, the need for clarity and a new ethics for such experiences will grow.

## Data Availability

N/A.

## Footnotes

1 Like any culturally constructed concept, the XR spectrum admits of a wide range of edge cases. In some sense of “simulated content” any picture or video can be said to be a simulation of reality and thus to present simulated content to a user. Following scholars like Liberati (2016) we restrict our conception of the XR spectrum to only those technologies that aim to digitally overlay content onto a user’s experience of the world (as AR devices like the HoloLens does) or to fully replace a user’s experience of the world with simulated content (as VR devices do). Digital filters fall within the XR spectrum, on our view, because they operate very much like AR devices: they are meant to replace an element of a user’s experience of the physical world with simulated content (e.g., filters that increase color saturation or remove blemishes both aim to replace an element of experience either by addition or subtraction). Televisions and computer screens, by contrast, are themselves a part of the user’s experience of the physical world (they aren’t overlaying content onto it in all but the thinnest sense). For more on the XR spectrum see (Milgram & Kishino, 1994; Tavinor, 2024).

2 In this paper we avoid claiming outright that experiences of XR embodiment are as *metaphysically* real as experiences of physical embodiment, though we do survey several theories of the self that would allow us to support that claim. For the purposes of this paper, it’s important that we show that the consequences (psychological and social) of XR embodiment merit further attention in the context of interpersonal, and especially intimate, friendships and relationships.

3 The idea behind embodied views of cognition is that our concepts are structured, at least in part, by how we evolved to sense the world. Concepts (abstract representational mental items) are thought to be dependent on reenactment of lower-level sensorimotor activity (Rizzolatti et al., 2001). For example, tokening the concept of an apple involves mirror-neuron activity of the sensorimotor cortex consistent with grabbing an apple. The view is not without critics (Caramazza et al., 2014). For our purposes, it’s enough to suggest that embodiment and cognition exercise at least a weak influence on one another.

4 Non-typical relationships between physical bodies and the self are possible. As we note below, though people may identify the self with their physical bodies this does not prevent them from modifying those physical bodies to better express that self using both cosmetic and surgical techniques. Rare conditions like Cotard’s Delusion which cause people to believe that they do not exist (Sahoo & Josephs, 2018) may sever (or at least alter) the connection between the self and embodiment.

5 Individuals technologically modify their physical bodies for all sorts of reasons, some of which others may endorse or reject. For the purposes of this paper, we don’t wish to take a stand on whether people *ought* to modify their bodies to, for example, conform to local aesthetic or gender norms. Instead, we claim that body modifications of this sort have a long history and scholarship about the ethics of such modifications is vast (Higgins & Wysong, 2018; McCabe et al., 2017; Sarajlic, 2020; Steensma & Cohen-Kettenis, 2018). In addition, XR technologies are already being used to carry out similar forms of body modification.

6 Transhumanist full-body cybernetic replacements, if possible at all, would impose their own physical limitations on embodiment though we ignore those here for simplicity.

7 One such restriction is that the users become dependent on corporations for their embodiment options and for continued access to their XR bodies (Won & Davis, 2023). The prospect of locking bodies that users strongly identify with behind microtransactions or membership fees has been largely unexplored though its implications are problematic.

8 Research into the ethics of XR embodiment is in its infancy. Some have argued that using XR to embody oneself in the bodies of others may be a form of harassment or even sexual deception (Ramirez et al., 2023). In the context of romantic relationships, the ethical dimension of XR embodiment is especially important.

9 Importantly, although both of Freeman’s subjects chose to create a unified identity by modifying their physical bodies to match their XR bodies, there remains the possibility that individuals can opt to create *blended* identities (that combine physical and XR bodies or that favor one form of embodiment in specific contexts). We explore such identities in greater detail in a later section of this article. See also Kim et al. (2023) for further research on the relationship between avatar appearance, physical appearance, and XR embodiment.

10 Zoom, for example, includes options to adjust many aspects of one’s appearance including both a “touch up my appearance” option (allowing users to cosmetically alter blemishes, wrinkles, etc.) and complete XR body overlays which have occasionally led to identity confusion (Li, 2021).

11 Similar proposals have been made for the inclusion of “Snapchat dysmorphia” (Ramphul & Mejias, 2018). Though it’s clear that conflicts between physical and XR embodiment may sometimes cause distress, it’s evident that they do not always do so and may even prove liberatory (as Freeman et al.’s, 2020 research suggests). For that reason we believe that pathologizing the conflict is, at best, only a partially accurate way of representing it.

12 Reidentification is a way of calling attention to the metaphysics of identity though, as noted, it is often motivated by normative concerns. In other words, philosophers care about whether the person in front of us calling herself Marika is the *same* Marika we met a decade ago because they care about moral questions relating to identity (if Marika owed us money then we can only ask the person in front of us to pay us back if she is *the same* person we lent money to).

13 For simplicity we ignore potential connections between reidentification and characterization (i.e., questions about whether substantial changes in who we take ourselves to be can change whether or not we are metaphysically the same person over time).

14 In other words, reductive or essentialist views of sex often attempt to define sex in terms of biological features (chromosomes, genes, neural structure, etc.) while choice and hybrid views often aim to define sex in terms of individual (or cultural) beliefs or judgments about biological features and the norms associated with them.

15 We say more about this below. As we argue, whether or not sexual orientation is understood as a biological or neurologically determined natural dyadic (or triadic) kind, a fluid continuum, a matter of choice, a social construction, or a combination of these, orientation itself is traditionally discussed in terms of a person’s orientation toward specific kinds of *physical* bodies.

16 Ayala (2017) argues, for example, for a conceptual act theory (Barrett, 2014) of sexual orientation where “in applying available conceptual resources, we figure out what our sexual-affective affects are, we make sense of them, without necessarily choosing amongst several alternative interpretations” (12). On such a view, one’s sexual orientation is a conceptual discovery but not a choice in the same way that naming one’s feelings with a specific emotion term (e.g., fear, disgust, etc.) is a conceptual discovery but which is bound by one’s cultural repertoire of emotional concepts.

17 We say more about XR embodiment and sex itself near the end of this article. While technologically mediated relationships have been discussed for some time (Ben-Ze’ev, 2004; Ley & Rambukkana, 2021), the distinct nature of immersive XR on relationships has not received similar attention.

18 Jennifer Haley, in her (2015) play *The Nether*, imagines a world where adults consensually engage in XR embodied sadomasochistic and pedophilic relationships with one another. It’s not clear that contemporary views on sexualized role play or sexual orientation can adequately describe just what it is that occurs in Haley’s play if we take XR embodiment seriously as a form of identity expansion or blending. However we choose to theorize it, ethical constraints should prevent individuals from engaging in these activities in a purely physically embodied form (i.e. it remains impossible for a person who is physically embodied as a child to morally consent to a sexual relationship).

19 It’s less clear to us how a view like Solomon’s would treat augmented reality body overlays in a sexual context. In such a context two individuals may be able to physically touch one another though what these individuals believe they are touching is likely to differ depending on whether we privilege physical embodiment or blended embodiment. When a lover desires to have sex with their beloved, how important is it for views like Solomon’s to depict their lover’s *physical* body accurately? If physical bodies are the aim of sex and if desiring to have sex with someone requires accurate representations of their physical body then even physical sex would not satisfy Solomon’s criteria if it is mediated by AR.

20 In earlier work, McArthur & Twist make this distinction clearer: “We draw a distinction between digisexualities, the technologies that allow for sexual experiences, and what we called digisexuals, people who see themselves as possessing a distinctive form of sexual identity. We can think of the distinction between those who merely use digisexual technologies on the one hand, and digisexuals as an identity-group on the other, according to analogies with other sexuality-based identities like those of an erotic orientation (e.g. kinky, fetishest, dominatrix, submissive, sadist, masochist, etc.), relational orientation (e.g. polyamorous, monogamous, polygamous, monogamish, etc.), and sexual orientation (e.g. gay, lesbian, bisexual, asexual, etc.). Many people engage in kinky, non-monogamous, or same-sex behaviour, but only a certain percentage of such people actually link their sexual identity to this behaviour” (McArthur & Twist, 2017). See also Ley and Rambukkana (2021) for a discussion of second wave digisexualities and their ethical issues.

21 Lloyd is aware of this dilemma. Near the end of her article she asks herself: “Does the framework I defend here place an undue burden on trans people to disclose private information about their sexuality and gender in order to discharge their duties of disclosure? This seems especially problematic in virtue of the fact that trans people already face undue burdens as a result of prejudice. I must admit that I am troubled by this implication of my view” (2021).

22 Scheidegger (2021) argues that labeling this practice as deceptive is, itself, morally problematic: “it might be argued that the characterization of a trans person’s behavior as active deception rests on the problematic or even false assumption that the categories sex and gender are fixed and therefore unchanging. Only within these parameters is it possible to argue that to live one’s life as a man, when one is biologically female, is a “deceptive” behavior.”

23 Both the Sam and José examples show us that Lloyd’s view of disclosure, and others like it, requires a person to undermine their identity when faced with someone who prioritizes the purely biological or physical elements of identity. While we do not here offer a solution to this dilemma about disclosure, we argue that the already-strained concepts of consent, identity, and sexual orientation require urgent attention as we enter an era where XR embodiment is likely to strain these concepts further.

24 Brogaard (2022) recently offered a counterargument to the view that sex by deception always vitiates consent. Deception itself can range from permissible to impermissible. Her claim is that consensual sex can be unethical despite being consensual and thus she contributes to the growing critique that consent to sex by itself makes sex permissible. Comparing cases of sex by deception, Brogaard argues that “[w]e are rarely (if ever) 100% honest with our sex partners—particularly not people we are about to have sex with for the first time (for fear that it may dampen their interest). Requiring ‘full revelation’ for consent to sex thus has the absurd implication that (nearly) all sex is rape. So, while sex by impersonation vitiates consent, sex by fake marital status does not” (Brogaard, 2022).

25 In the USA Institutional Review Boards (IRBs) must abide by the Code of Federal Regulations pertaining to research ethics. We here specifically refer to 45 CFR 46 § 46.111 (2009).

26 Lisa Nakamura’s work on “Cybertpes” (2002) and ethnicity in VR (2020) expose some of the challenges associated with research on identity in online and XR spaces. Nakamura argues against using XR embodiment as a way of enhancing empathy. She claims that such projects are misguided and XR embodiment like this becomes “toxic” by “occupying the body of an other who might not even own their own body” (2020). See also Ramirez et al., (2021) for an argument that VR perspective taking is psychologically implausible and often unethical.

27 Embodying someone as a cow on the way to a slaughterhouse or in the bodies of people of races, genders, or ethnicities different from those of the subject raise clear ethical issues both for those being subjected to the experience (e.g., it may be traumatic to be embodied as a cow about to slaughtered) but also for those whose bodies are being used as props in the simulation (e.g., ethical issues with using the bodies of minority populations as things to embody others within).
